# Tobacco smoking and the risk of aortic aneurysm in the UK biobank

**DOI:** 10.1038/s41598-025-18013-x

**Published:** 2025-09-01

**Authors:** Lili Zheng, Ghaliah Baroom, Rida E. Z. Naqvi, Alicia K. Heath, Antonio Berlanga-Taylor, Makoto Hibino, Dagfinn Aune

**Affiliations:** 1 Department of Epidemiology and Biostatistics, School of Public Health, Imperial College London, White City Campus, 90 Wood Lane, London, W12 0BZ UK; 2 Unidad de Medicina de Datos, Centro Médico Nacional Siglo XXI, Instituto Mexicano del Seguro Social, Mexico City, Mexico; 3 Department of Thoracic and Cardiovascular Surgery, Heart, Vascular and Thoracic Institute, Cleveland Clinic, Cleveland, OH USA; 4 Department of Nutrition, Oslo New University College, Oslo, Norway; 5 Department of Research, Cancer Registry of Norway, Norwegian Institute of Public Health, Oslo, Norway

**Keywords:** Tobacco smoking, Aortic aneurysm, Prospective study, Cardiovascular diseases, Cardiology, Diseases, Risk factors

## Abstract

Smoking is an established risk factor for aortic aneurysm, however, few studies have investigated the associations with different aortic aneurysm subsites. The aim of this study was to investigate whether different tobacco smoking metrics and smoking cessation was associated with aortic aneurysm and its subsites in the UK Biobank. The analysis included 495,993 participants, aged 37–73 years. Cox proportional hazards models were used to estimate multivariable-adjusted hazard ratios (HRs) and 95% confidence intervals (CIs) for the association between smoking and aortic aneurysm. After 12.3 years of follow-up, 3,353 incident cases and 184 deaths from aortic aneurysm were identified. The HRs (95% CIs) of aortic aneurysm incidence for current and former vs. never smokers were 4.32 (3.93–4.76) and 1.70 (1.56–1.84), respectively, and for ≥ 20 vs. <10 cigarettes per day was 5.67 (4.93–6.52), and similar results were observed for pack-years and duration of smoking. At ≥ 30 years of smoking cessation vs. current smoking risk was substantially reduced (0.23, 0.20–0.27) and comparable to never smoking (0.22, 0.20–0.25). Strong positive HRs were observed for current vs. never smoking and abdominal (8.90, 7.79–10.16), thoracoabdominal (11.64, 4.20-32.25), and unspecified site (2.06, 1.61–2.65), ruptured (10.47, 6.12–17.90) and non-ruptured (4.19, 3.80–4.62) aortic aneurysms, but no association was observed for thoracic aortic aneurysm (1.13, 0.88–1.44). Smoking was strongly associated with increased risk of aortic aneurysm and most subtypes. Smoking cessation for ≥ 30 years strongly reduced risk to levels comparable of never smokers. These findings support public health policies promoting smoking prevention and cessation.

**Supplementary Information:**

The online version contains supplementary material available at 10.1038/s41598-025-18013-x.

## Introduction

Globally, aortic aneurysm accounted for 167,200 deaths and 3 million years-of-life-lost in 2017^1^. At least 80% of patients with aortic aneurysm rupture die before reaching a hospital^2^. However, most aneurysms are asymptomatic before they rupture^3,4^. Risk factors for aortic aneurysm include older age^1^, male sex^2^, connective tissue diseases (Ehlers-Danlos and Marfan’s syndrome)^3^, high blood pressure^4^, low physical activity^5^, and smoking^6^, while diabetes is associated with lower risk^7^.

A large number of studies have examined the association between tobacco smoking status and risk of aortic aneurysm^8–11^ or abdominal aortic aneurysm^11–14^. Fewer studies have been published on cigarettes per day^15–18^ and pack-years^9,12,19,20^, however, these have in general also been consistent in showing an increased risk. Studies have also been relatively consistent in showing an inverse association between time since quitting smoking and risk of aortic aneurysm, however, again the number of studies available is limited^10,12,21–23^. Few studies have assessed the association between smoking and aortic aneurysm subtypes, such as abdominal vs. thoracic vs. thoracoabdominal aneurysms or ruptured vs. non-ruptured aneurysms. Although men have a higher incidence of aortic aneurysm than women, women have similar or higher rates of aneurysm rupture^24,25^. Aortic aneurysm rupture is known to increase fatality rate, but few population-based studies have investigated the association between smoking and aortic aneurysm rupture, and most previous studies were patient-based studies or had a small number of cases of ruptured aortic aneurysms^25–27^.

We therefore analysed the associations between different measures of smoking and smoking cessation and risk of aortic aneurysm incidence and mortality, overall and by aortic aneurysm subsites as well as ruptured vs. non-ruptured aneurysms in the UK Biobank, a cohort study of 0.5 million UK adults.

## Methods

### Study population

The UK Biobank is a prospective cohort study of 0.5 million UK adults aged 37–73 at recruitment between March 2006 and August 2010^28^. Participants were recruited across 21 assessment centers in England, Wales and Scotland and provided written informed consent to participate. Information about diet, smoking, alcohol intake, socio-demographic factors, medical history was collected via touchscreen questionnaires, and a clinical examination including measurement of anthropometric factors, blood pressure and a blood sample was undertaken.

The current dataset included 502,359 participants, and we excluded in subsequent order persons with prevalent aortic aneurysm at baseline (*n* = 476), different self-reported vs. genetic sex (*n* = 372), missing data on BMI (*n* = 3,096), and smoking status (*n* = 2,422). After exclusion, a total of 495,993 participants (225,637 men and 270,356 women) were included in the statistical analysis.

### Assessment of smoking and covariates

Data on smoking, alcohol intake, physical activity, socio-demographic characteristics, and medical history were collected using touchscreen questionnaires, and weight and height data were measured. Physical activity was categorized as the sum of frequencies of walking, moderate and vigorous physical activity per week, with each episode lasting at least 10 min. For the current analysis, we used information on smoking status, number of cigarettes smoked per day, pack-years, age at starting smoking, duration of smoking, and time since quitting smoking as the main exposures.

### Outcome assessment

Incident cases of aortic aneurysm were identified by linkages to Hospital Episode Statistics for England (HES), Scottish Morbidity Records (SMR), and the Patient Database for Wales. Participants with aortic aneurysm listed as underlying cause of death were identified by linkages to National Health Service (NHS) England (for England and Wales) and NHS Central Register, National Records of Scotland. Aortic aneurysm cases were identified according to the 10th revision of the International Statistical Classification of Diseases (ICD-10) diagnostic codes I71.1-I71.9 and ICD-9 codes 441.1-441.9. Aortic aneurysm subtypes were classified as follows: thoracic aortic aneurysm (I71.1-I71.2), abdominal aortic aneurysm (I71.3-I71.4), thoracoabdominal aortic aneurysm (I71.5-I71.6), and unspecified site aortic aneurysm (I71.8-I71.9), ruptured aortic aneurysm (I71.1, I71.3, I71.5, I71.8), and non-ruptured aortic aneurysm (I71.2, I71.4, I71.6, I71.9). For participants with aortic aneurysm listed as underlying cause of death, but with no previous hospital diagnosis of aortic aneurysm we used date of death as diagnosis date. Participants were followed up from the date of recruitment until the date of aortic aneurysm diagnosis, death, loss to follow-up, or the last complete follow-up (30 September 2021 for England and Wales and 31 October 2021 for Scotland), whichever occurred first. The number of participants lost to follow-up over the study period was low (*n* = 1,275, 0.26%).

### Statistical analysis

Multivariable Cox proportional hazards regression models were applied to estimate hazard ratios (HRs) and 95% confidence intervals (CIs) for the associations between smoking status, cigarettes per day, pack-years, duration, age started, and years since quitting smoking and the risk of aortic aneurysm. The multivariable model included age (continuous), sex (men vs. women), ethnicity (white, non-white), Townsend deprivation index (quintiles), education (quintiles), body mass index (BMI, categories), height (sex-specific quintiles), frequency of leisure-time physical activity (quintiles, times per week), history of connective tissue disease (Marfan syndrome or Ehlers-Danlos syndrome) (yes vs. no), and each smoking variable (categorical) one at a time. Missing data on the confounders of interest was low (0.12%-1.8%), except for physical activity (8.8%), and were handled as a missing category indicator in the models. Persons with missing data on each smoking variable were excluded from the respective analyses and therefore the number of participants included differs across the different smoking metrics. We did not adjust for hypertension or blood pressure in the models, to avoid over-adjustment for a potential intermediate factor, because smoking is associated with an increased risk of hypertension^29,30^. Tests for linear trends across categories of smoking variables were conducted by generating a new variable where the original smoking variable had the categories replaced by the median within each category, and by entering this variable as a continuous variable in the model. Subgroup analyses were conducted stratified by age, sex, hypertension and BMI to investigate potential effect modification and a Wald test was used to test for interactions. All analyses were conducted using Stata version 16.1 (StataCorp, College Station, TX, USA).

## Results

A total of 495,993 participants were included in the analytical dataset. When compared to never smokers, a higher proportion of current smokers were women than men. In addition, current smokers were younger, more deprived, and had lower education levels. We found few differences in smoking prevalence by ethnicity, physical activity, body mass index, height and connective tissue disease (Table 1). During an average of 12.3 years (6.1 million person-years) follow-up, a total of 3,353 incident aortic aneurysm cases were identified, including 778 thoracic, 1,985 abdominal, 26 thoracoabdominal and 519 unspecified aortic aneurysms. Of the total, 121 ruptured and 3,232 were non-ruptured aortic aneurysm cases, and 184 aortic aneurysm deaths occurred.

**Table 1:** Population characteristics by smoking status.

		Never	Former	Current
N		271,750	171,901	52,342
Age, years (median)		57	60	55
Sex	Men (%)	59.3	49.5	46.1
	Women (%)	40.7	50.5	53.9
Ethnicity	White (%)	93.0	96.8	93.5
	Non-white (%)	6.7	2.9	6.0
	Missing (%)	0.3	0.3	0.5
Townsend Deprivation Index (highest quintile, %)		16.6	19.8	36.1
Education (highest category, %)		32.2	35.8	22.7
Physical activity (highest category, %)		15.6	16.4	15.8
Body mass index, kg/m^2^ (median)		26.4	27.3	26.5
Height in men/women, cm (median)		176/162	175/163	175/162
Connective tissue disease (%)		0.05	0.04	0.05

### Smoking and aortic aneurysm

The hazard ratios (95% CIs) of aortic aneurysm overall for current, former and ever vs. never smokers were 4.32 (3.93–4.76), 1.70 (1.56–1.84), and 2.15 (1.99–2.32), respectively. There was a strong dose-response relationship between increasing number of cigarettes per day and aortic aneurysm with HRs (95% CIs) of 3.62 (2.84–4.62), 5.66 (4.93–6.50), and 5.67 (4.93–6.52) for < 10, 10-<20 and ≥ 20 cigarettes per day vs. never smokers and a significant linear trend (p_trend_ = < 0.001). Pack-years of smoking was positively associated with aortic aneurysm risk with HRs (95% CIs) of 1.37 (1.18–1.60), 1.64 (1.44–1.88), 2.47 (2.19–2.80), and 3.77 (3.43–4.14; p_trend_ = < 0.001) for < 10, 10-<20, 20-<30, and ≥ 30 pack-years vs. never smokers. Similarly, greater duration of smoking was associated with dose-dependent increases in risk among current smokers and former smokers, with HRs (95% CIs) 2.52 (1.66–3.84), 3.48 (2.78–4.35), 5.86 (5.24–6.56; p_trend_ = < 0.001) for < 10, 10-<20, and ≥ 20 years in current smokers and 1.09 (0.95–1.24), 1.61 (1.40–1.84), and 3.18 (2.87–3.53; p_trend_ = < 0.001) for the same comparison in former smokers. Younger age (< 16 vs. ≥16 years) at starting smoking was slightly more strongly associated with aortic aneurysm both in former smokers (2.11, 1.87–2.37 vs. 1.79, 1.62–1.96) and current smokers (5.62, 4.87–6.47 vs. 4.82, 4.29–5.42). Years since quitting smoking was associated with a dose-dependent reduction in risk of aortic aneurysm with HRs (95% CIs) of 0.77 (0.69–0.87), 0.50 (0.44–0.57), 0.32 (0.28–0.37), and 0.23 (0.20–0.27; p_trend_ = < 0.001) for < 10, 10-<20, 20-<30, ≥ 30 years vs. current smoking, and which at ≥ 30 years duration approached that of never smokers (0.22, 0.20–0.25) (Table 2).

**Table 2:** Smoking and aortic aneurysm.

			Aortic aneurysm	
	Participants	Person-years	Cases	HR (95% CI)
Smoking status				
Never	271,750	3370939.3	955	1.00
Former	171,901	2095054.6	1566	1.70 (1.56–1.84)
Current	52,342	627236.9	832	4.32 (3.93–4.76)
Ever	224,243	2722291.5	2398	2.15 (1.99–2.32)
Cigarettes/day				
Never	271,750	3370939.3	955	1.00
Former	171,901	2095054.6	1566	1.69 (1.56–1.84)
< 10	7146	86774.6	70	3.62 (2.84–4.62)
10-<20	15,225	182485.4	268	5.66 (4.93–6.50)
≥ 20	13,313	156134.7	267	5.67 (4.93–6.52)
p_trend_				< 0.001
Pack-years				
Never	271,750	3370939.3	955	1.00
< 10	37,410	461046.2	196	1.37 (1.18–1.60)
10-<20	40,372	494026.0	295	1.64 (1.44–1.88)
20-<30	29,125	353087.3	348	2.47 (2.19–2.80)
≥ 30	42,679	496908.6	992	3.77 (3.43–4.14)
p_trend_				< 0.001
Duration - current				
Never	271,750	3370939.3	955	1.00
< 10	8463	105480.8	24	2.52 (1.66–3.84)
10-<20	13,319	162785.5	95	3.48 (2.78–4.35)
≥ 20	16,483	187198.4	564	5.86 (5.24–6.56)
p_trend_				< 0.001
Duration - former				
Never	271,750	3370939.3	955	1.00
< 10	51,805	637845.3	279	1.09 (0.95–1.24)
10-<20	30,144	367360.6	272	1.61 (1.40–1.84)
≥ 20	32,029	376846.9	694	3.18 (2.87–3.53)
p_trend_				< 0.001
Age started				
Never	271,750	3370939.3	955	1.00
< 16 years, former	33,669	406128.8	438	2.11 (1.87–2.37)
≥ 16	80,765	981347.2	815	1.79 (1.62–1.96)
< 16 years, current	13,025	153502.7	265	5.62 (4.87–6.47)
≥ 16	25,240	301962.0	418	4.82 (4.29–5.42)
Years since quit				
Current	52,342	627236.9	832	1.00
< 10	31,312	377222.5	463	0.77 (0.69–0.87)
10-<20	26,649	323076.3	308	0.50 (0.44–0.57)
20-<30	30,623	373732.0	263	0.32 (0.28–0.37)
≥ 30	25,922	314323.9	218	0.23 (0.20–0.27)
Never	271,750	3370939.3	955	0.22 (0.20–0.25)
p_trend_				< 0.001

Adjusted for age, sex, ethnicity, Townsend deprivation index, education, BMI, height, leisure-time physical activity, history of connective tissue disease.

### Smoking and aortic aneurysm subsites (thoracic, abdominal, thoracoabdominal and unspecified site)

When analyses were conducted separately for thoracic, abdominal, thoracoabdominal and unspecified site aortic aneurysms, strong positive associations were observed for current vs. never smokers and abdominal (8.90, 7.79–10.16), thoracoabdominal (11.64, 4.20-32.25), and unspecified site (2.06, 1.61–2.65) aortic aneurysms, but no clear association was observed for thoracic aortic aneurysm (1.13, 0.88–1.44), and there was significant heterogeneity by subsite (p_heterogeneity_<0.0001) (Table 3). Similar trends were observed for ≥ 20 cigarettes/day vs. never smoking for abdominal (12.32, 10.34–14.68), thoracoabdominal (21.59, 6.14–75.96), and unspecified site (2.48, 1.65–3.72), and thoracic (0.99, 0.60–1.62) aortic aneurysms (p_heterogeneity_<0.0001), and for ≥ 30 pack-years vs. never smoking for abdominal (7.24, 6.35–8.26), thoracoabdominal (8.02, 2.73–23.55), unspecified site (1.63, 1.26–2.10), and thoracic (1.17, 0.93–1.48) aortic aneurysms (p_heterogeneity_<0.0001). Consistent with these findings, ≥ 20 years duration of smoking among current smokers vs. never smoking was positively associated with abdominal aortic aneurysm (11.47, 9.89–13.30), thoracoabdominal (11.88, 3.53–39.92), unspecified site (2.51, 1.84–3.44), but not thoracic (1.25, 0.88–1.77) aortic aneurysm (p_heterogeneity_<0.0001), and for the same comparison in former smokers positive associations were observed for abdominal (5.76, 5.00-6.63), thoracoabdominal (4.70, 1.40-15.79), unspecified site (1.48, 1.12–1.97), but not thoracic (1.06, 0.82–1.37) aortic aneurysm (p_heterogeneity_<0.0001). Younger age at starting smoking (< 16 vs. ≥16 years) was more strongly positively associated with abdominal aortic aneurysm both in former (3.72, 3.18–4.35 vs. 2.97, 2.59–3.40) and current (12.28, 10.31–14.64 vs. 10.04, 8.61–11.71) smokers when compared to never smokers, while for thoracoabdominal (8.64, 1.59–46.86 vs. 9.80, 2.88–33.28), and unspecified site (2.28, 1.50–3.46 vs. 2.19, 1.58–3.03), the associations were limited to current smokers and no association was observed for thoracic aortic aneurysms (p_heterogeneity_<0.0001 for both former and current smokers). Longer duration of quitting smoking was inversely associated with risk of abdominal aortic aneurysm (0.14, 0.11–0.17 for ≥ 30 years), thoracoabdominal (0.12, 0.02–0.97 for 10-<20 years), and unspecified site (0.46, 0.32–0.68), but not thoracic (0.89, 0.64–1.24) aortic aneurysms (p_heterogeneity_<0.0001) (Table 3).

**Table 3:** Smoking and aortic aneurysm subtypes.

			Thoracic aortic aneurysm		Abdominal aortic aneurysm		Thoracoabdominal aortic aneurysm		Unspecified aortic aneurysm	
	Participants	Person-years	Cases	HR (95% CI)	Cases	HR (95% CI)	Cases	HR (95% CI)	Cases	HR (95% CI)
Smoking status										
Never	271,750	3370939.3	381	1.00	305	1.00	6	1.00	218	1.00
Former	171,901	2095054.6	317	1.02 (0.88–1.19)	1032	2.77 (2.45–3.13)	8	1.68 (0.57–4.94)	209	1.07 (0.88–1.30)
Current	52,342	627236.9	80	1.13 (0.88–1.44)	648	8.90 (7.79–10.16)	12	11.64 (4.20-32.25)	92	2.06 (1.61–2.65)
Ever	224,243	2722291.5	397	1.04 (0.90–1.20)	1680	3.77 (3.36–4.24)	20	3.45 (1.35–8.79)	301	1.25 (1.05–1.50)
p_heterogeneity−by−subsite_				< 0.0001						
Cigarettes/day										
Never	271,750	3370939.3	381	1.00	350	1.00	6	1.00	218	1.00
Former	171,901	2095054.6	317	1.02 (0.87–1.19)	1032	2.76 (2.45–3.13)	8	1.67 (0.57–4.92)	209	1.07 (0.88–1.30)
< 10	7146	86774.6	8	1.00 (0.50–2.02)	54	7.81 (5.86–10.41)	3	21.54 (5.28–87.81)	5	1.08 (0.44–2.62)
10-<20	15,225	182485.4	27	1.49 (1.01–2.22)	213	12.19 (10.25–14.51)	1	3.44 (0.40-29.25)	27	2.40 (1.60–3.61)
≥ 20	13,313	156134.7	17	0.99 (0.60–1.62)	217	12.32 (10.34–14.68)	5	21.59 (6.14–75.96)	28	2.48 (1.65–3.72)
p_trend_				< 0.0001		< 0.001		< 0.001		< 0.001
p_heterogeneity−by−subsite_				< 0.0001						
Pack-years										
Never	271,750	3370939.3	381	1.00	350	1.00	6	1.00	218	1.00
< 10	37,410	461046.2	51	0.92 (0.69–1.23)	104	1.94 (1.56–2.42)	2	2.59 (0.52–12.94)	39	1.20 (0.85–1.69)
10-<20	40,372	494026.0	71	1.09 (0.84–1.41)	184	2.64 (2.21–3.16)	1	1.08 (0.13–9.02)	39	0.98 (0.70–1.38)
20-<30	29,125	353087.3	44	0.90 (0.65–1.23)	260	4.68 (3.98–5.50)	1	1.41 (0.17–11.91)	43	1.38 (0.99–1.93)
≥ 30	42,679	496908.6	97	1.17 (0.93–1.48)	793	7.24 (6.35–8.26)	10	8.02 (2.73–23.55)	92	1.63 (1.26–2.10)
p_trend_				0.201		< 0.001		< 0.001		< 0.001
p_heterogeneity−by−subsite_				< 0.0001						
Duration - current										
Never	271,750	3370939.3	381	1.00	350	1.00	6	1.00	218	1.00
< 10	8463	105480.8	8	1.55 (0.75–3.20)	13	5.69 (3.22–10.05)	0	-	3	1.06 (0.33–3.40)
10-<20	13,319	162785.5	16	1.23 (0.74–2.06)	65	8.59 (6.44–11.44)	1	12.90 (1.32-126.38)	13	1.74 (0.97–3.11)
≥ 20	16,483	187198.4	37	1.25 (0.88–1.77)	466	11.47 (9.89–13.30)	6	11.88 (3.53–39.92)	55	2.51 (1.84–3.44)
p_trend_				0.111		< 0.001		< 0.001		< 0.001
p_heterogeneity−by−subsite_				< 0.0001						
Duration - former										
Never	271,750	3370939.3	381	1.00	350	1.00	6	1.00	218	1.00
< 10	51,805	637845.3	86	0.93 (0.74–1.18)	134	1.30 (1.07–1.59)	0	-	59	1.04 (0.78–1.39)
10-<20	30,144	367360.6	64	1.13 (0.86–1.47)	178	2.51 (2.09–3.02)	0	-	30	0.82 (0.55–1.20)
≥ 20	32,029	376846.9	73	1.06 (0.82–1.37)	546	5.76 (5.00-6.63)	6	4.70 (1.40-15.79)	69	1.48 (1.12–1.97)
p_trend_				0.521		< 0.001		0.024		0.047
p_heterogeneity−by−subsite_				< 0.0001						
Age started										
Never	271,750	3370939.3	381	1.00	350	1.00	6	1.00	218	1.00
< 16 years, former	33,669	406128.8	59	0.92 (0.69–1.21)	321	3.72 (3.18–4.35)	3	3.17 (0.74–13.67)	55	1.22 (0.90–1.66)
≥ 16	80,765	981347.2	165	1.07 (0.89–1.29)	544	2.97 (2.59–3.40)	3	1.21 (0.30–4.96)	103	1.05 (0.83–1.33)
< 16 years, current	13,025	153502.7	16	0.96 (0.58–1.60)	221	12.28 (10.31–14.64)	2	8.64 (1.59–46.86)	26	2.28 (1.50–3.46)
≥ 16	25,240	301962.0	45	1.37 (1.00-1.87)	323	10.04 (8.61–11.71)	5	9.80 (2.88–33.28)	45	2.19 (1.58–3.03)
p_heterogeneity−by−subsite_				< 0.0001						
Years since quit										
Current	52,342	627236.9	80	1.00	648	1.00	12	1.00	92	1.00
< 10	31,312	377222.5	54	1.00 (0.70–1.41)	353	0.73 (0.64–0.83)	5	0.64 (0.22–1.83)	51	0.81 (0.57–1.14)
10-<20	26,649	323076.3	47	0.89 (0.62–1.27)	230	0.45 (0.38–0.52)	1	0.12 (0.02–0.97)	30	0.48 (0.32–0.73)
20-<30	30,623	373732.0	58	0.84 (0.59–1.18)	166	0.24 (0.21–0.29)	0	-	39	0.49 (0.33–0.71)
≥ 30	25,922	314323.9	65	0.89 (0.64–1.24)	113	0.14 (0.11–0.17)	0	-	40	0.46 (0.32–0.68)
Never	271,750	3370939.3	381	0.87 (0.68–1.12)	350	0.11 (0.09–0.12)	6	0.08 (0.03–0.23)	218	0.49 (0.38–0.63)
p_trend_				0.205		< 0.001		0.004		< 0.001
p_heterogeneity−by−subsite_				< 0.0001						

Adjusted for age, sex, ethnicity, Townsend deprivation index, education, BMI, height, leisure-time physical activity, history of connective tissue disease.

### Smoking and ruptured and non-ruptured aortic aneurysm

The HR (95% CIs) for current vs. never smokers was 10.47 (6.12–17.90) for ruptured aortic aneurysm and 4.19 (3.80–4.62) for non-ruptured aortic aneurysm (p_heterogeneity_=0.01) (Table 4). The HRs of ruptured and non-ruptured aortic aneurysm relative to never smokers were 13.29 (6.40-27.61) and 5.51 (4.77–6.36) for ≥ 20 cigarettes/day (p_heterogeneity_=0.02), 7.51 (4.40-12.84) and 3.68 (3.35–4.05) for ≥ 30 pack-years (p_heterogeneity_=0.01), 10.12 (5.47–18.71) and 5.77 (5.14–6.47) for ≥ 20 years duration in current smokers (p_heterogeneity_=0.08), 6.36 (3.63–11.14) and 3.10 (2.79–3.44) for ≥ 20 years duration in former smokers (p_heterogeneity_=0.01), and 4.96 (2.68–9.16) and 2.04 (1.81–2.30) for < 16 years age at starting smoking in former smokers and 15.65 (7.73–31.71) vs. 5.41 (4.68–6.25) for < 16 years age at starting smoking in current smokers (p_heterogeneity_=0.008), respectively. The HRs for ≥ 30 years of smoking cessation vs. current smoking was 0.10 (0.04–0.23) for ruptured and 0.24 (0.21–0.26) for non-ruptured aortic aneurysm (p_heterogeneity_=0.002) (Table 4).

**Table 4:** Smoking and ruptured aortic aneurysm, non-ruptured aortic aneurysm and aortic aneurysm mortality.

			Ruptured aortic aneurysm		Non-ruptured aortic aneurysm		Aortic aneurysm mortality		
	Participants	Person-years	Cases	HR (95% CI)	Cases	HR (95% CI)	Person-years	Cases	HR (95% CI)
Smoking status									
Never	271,750	3370939.3	21	1.00	934	1.00	3,374,710	35	1.00
Former	171,901	2095054.6	58	2.66 (1.60–4.40)	1508	1.67 (1.54–1.82)	2,101,463	83	2.22 (1.49–3.31)
Current	52,342	627236.9	42	10.47 (6.12–17.90)	790	4.19 (3.80–4.62)	630275.2	66	8.78 (5.75–13.38)
Ever	224,243	2722291.5	100	3.86 (2.39–6.21)	2298	2.11 (1.95–2.28)	2731738.2	149	3.31 (2.27–4.81)
p_heterogeneity−by−subtype_				0.01			6106448.2		
Cigarettes/day									
Never	271,750	3370939.3	21	1.00	934	1.00	3,374,710	35	1.00
Former	171,901	2095054.6	58	2.64 (1.60–4.38)	1508	1.67 (1.54–1.82)	2,101,463	83	2.21 (1.48–3.30)
< 10	7146	86774.6	4	9.60 (3.28–28.07)	66	3.49 (2.71–4.48)	87013.7	7	9.57 (4.23–21.65)
10-<20	15,225	182485.4	17	17.32 (9.01–33.28)	251	5.41 (4.70–6.24)	183563.9	24	12.97 (7.61–22.09)
≥ 20	13,313	156134.7	12	13.29 (6.40-27.61)	255	5.51 (4.77–6.36)	157051.1	22	11.94 (6.86–20.79)
p_trend_				< 0.001		< 0.001			< 0.001
p_heterogeneity−by−subtype_				0.02					
Pack-years									
Never	271,750	3370939.3	21	1.00	934	1.00	3,374,710	35	1.00
< 10	37,410	461046.2	8	2.44 (1.08–5.51)	188	1.35 (1.15–1.58)	461843.2	14	2.64 (1.42–4.91)
10-<20	40,372	494026.0	9	2.17 (0.99–4.75)	286	1.63 (1.43–1.87)	495172.5	9	1.33 (0.64–2.77)
20-<30	29,125	353087.3	14	4.33 (2.19–8.56)	334	2.43 (2.14–2.76)	354489.4	25	4.57 (2.72–7.68)
≥ 30	42,679	496908.6	46	7.51 (4.40-12.84)	946	3.68 (3.35–4.05)	500792.2	79	7.09 (4.67–10.76)
p_trend_				< 0.001		< 0.001			< 0.001
p_heterogeneity−by−subtype_				0.01					
Duration - current									
Never	271,750	3370939.3	21	1.00	934	1.00	3,374,710	35	1.00
< 10	8463	105480.8	2	19.51 (4.47–85.20)	22	2.30 (1.48–3.56)	105568.5	3	15.74 (4.71–52.62)
10-<20	13,319	162785.5	8	26.27 (10.48–65.84)	87	3.19 (2.53–4.02)	163094.2	6	9.39 (3.69–23.91)
≥ 20	16,483	187198.4	24	10.12 (5.47–18.71)	540	5.77 (5.14–6.47)	189267.1	50	10.95 (6.90-17.36)
p_trend_				< 0.001		< 0.001			< 0.001
p_heterogeneity−by−subtype_				0.08					
Duration - former									
Never	271,750	3370939.3	21	1.00	934	1.00	3,374,710	35	1.00
< 10	51,805	637845.3	4	0.63 (0.22–1.85)	275	1.10 (0.96–1.26)	639068.9	13	1.31 (0.69–2.49)
10-<20	30,144	367360.6	7	1.62 (0.68–3.84)	265	1.61 (1.40–1.85)	368528.4	11	1.52 (0.76–3.02)
≥ 20	32,029	376846.9	37	6.36 (3.63–11.14)	657	3.10 (2.79–3.44)	379673.4	49	4.75 (3.01–7.51)
p_trend_				< 0.001		< 0.001			< 0.001
p_heterogeneity−by−subtype_				0.01					
Age started									
Never	271,750	3370939.3	21	1.00	934	1.00	3,374,710	35	1.00
< 16 years, former	33,669	406128.8	23	4.96 (2.68–9.16)	415	2.04 (1.81–2.30)	408033.2	31	3.58 (2.17–5.92)
≥ 16	80,765	981347.2	25	2.18 (1.21–3.93)	790	1.78 (1.62–1.96)	984689.4	42	2.24 (1.42–3.53)
< 16 years, current	13,025	153502.7	14	15.65 (7.73–31.71)	251	5.41 (4.68–6.25)	154506.3	24	12.73 (7.38–21.98)
≥ 16	25,240	301962.0	20	11.01 (5.90-20.52)	398	4.69 (4.16–5.28)	303423.6	35	10.33 (6.40-16.66)
p_heterogeneity−by−subtype_				0.008					
Years since quit									
Current	52,342	627236.9	42	1.00	790	1.00	630275.2	66	1.00
< 10	31,312	377222.5	26	0.82 (0.50–1.34)	437	0.77 (0.69–0.87)	379052.9	35	0.73 (0.48–1.11)
10-<20	26,649	323076.3	11	0.32 (0.16–0.62)	297	0.51 (0.44–0.58)	324364.8	16	0.31 (0.18–0.55)
20-<30	30,623	373732.0	5	0.11 (0.04–0.27)	258	0.34 (0.29–0.39)	374848.6	9	0.14 (0.07–0.28)
≥ 30	25,922	314323.9	6	0.10 (0.04–0.23)	212	0.24 (0.21–0.28)	315320.9	13	0.16 (0.09–0.30)
Never	271,750	3370939.3	21	0.09 (0.05–0.16)	934	0.23 (0.21–0.26)	3,374,710	35	0.11 (0.07–0.17)
p_trend_				< 0.001		< 0.001			< 0.001
p_heterogeneity−by−subtype_				0.002					

Adjusted for age, sex, ethnicity, Townsend deprivation index, education, BMI, height, leisure-time physical activity, history of connective tissue disease.

### Smoking and aortic aneurysm mortality

The HR (95% CIs) for current vs. never smokers was 8.78 (5.75–13.38) for aortic aneurysm mortality (Table 4). The HRs of aortic aneurysm mortality relative to never smokers were 11.94 (6.86–20.79) for ≥ 20 cigarettes/day, 7.09 (4.67–10.76) for ≥ 30 pack-years, 10.95 (6.90-17.36) for ≥ 20 years duration in current smokers, 4.75 (3.01–7.51) for ≥ 20 years duration in former smokers, and 3.58 (2.17–5.92) and 12.73 (7.38–21.98) for < 16 years age at starting smoking in former and current smokers, respectively. The HR for ≥ 30 years of smoking cessation vs. current smoking was 0.16 (0.09–0.30) for aortic aneurysm mortality (Table 4).

### Stratified analyses and sensitivity analyses

In stratified analyses, interactions were observed when the analyses of smoking status were stratified by age (*p* < 0.001), sex (*p* < 0.001), BMI (*p* = 0.002), but not for hypertension (*p* = 0.89) (Supplementary Table 1). Stronger associations for current vs. never smokers were observed among older vs. younger participants (≥ 60 vs. <60 years) (5.13, 4.58–5.75 vs. 2.84, 2.37–3.41), for men vs. women (5.43, 4.47–6.60 vs. 4.09, 3.67–4.57), for those with normal BMI vs. those with overweight or obesity (5.39, 4.48–6.48 vs. 4.13, 3.58–4.75 vs. 3.81, 3.17–4.59), but associations were similar in those with vs. without hypertension (4.32, 3.86–4.84 vs. 4.45, 3.71–5.34) (Supplementary Table 1).

Further adjustment for prevalent bicuspid aortic valve at baseline did not alter the results of the main analysis (results not shown).

## Discussion

In this large-scale cohort study, we found strong positive associations between smoking status, cigarettes/day, pack-years, longer duration, and younger age at starting smoking and risk of aortic aneurysm overall with 3- to 6-fold increases in risk. When specific subsites and subtypes were investigated strong positive associations were observed for abdominal, thoracoabdominal, and unspecified site aortic aneurysms, ruptured and non-ruptured aortic aneurysm, and for aortic aneurysm mortality. In contrast, little evidence of an association was observed for thoracic aortic aneurysm. Longer duration of smoking cessation substantially reduced the risk of aortic aneurysm compared to current smokers and risk approached that of never smokers at ≥ 30 years of smoking cessation.

The strong positive associations observed between tobacco smoking and the risk of aortic aneurysm is consistent with a large body of epidemiological evidence that has consistently reported this finding^6,15–18^. The findings are consistent with a recent meta-analysis of 23 prospective studies, which found a 4.9-fold increased risk of aortic aneurysm among current vs. never smokers, a 6-fold increased risk with 20 cigarettes smoked per day vs. none and a 6–7 fold increase in risk at 20–25 pack-years vs. none, and a substantial reduction in risk with around 30 years of smoking cessation when compared to current smokers^6^. Our findings are consistent with a recent Swedish study which reported odds ratios (95% CIs) of 7.9 (5.3–11.7) for current vs. never smokers in relation to abdominal aortic aneurysm and of 1.3 (0.8–1.9) for the same comparison in relation to thoracic aortic aneurysm^31^. However, we are not aware of previous studies that have reported on the association between smoking and thoracoabdominal aortic aneurysms. This study is also one of the first to report separately on ruptured vs. non-ruptured aortic aneurysms, and the association appeared to be stronger with the ruptured than the non-ruptured aneurysms, and this likely contributes to the stronger associations we observed with aortic aneurysm mortality than for incidence. Similarly, a substantial relative risk reduction of ~ 90% of ruptured aortic aneurysm was observed with 20 to 30 or more years of smoking cessation, and risk was comparable to that of never smokers with such a duration of smoking cessation. In the current analysis, there was a slightly stronger association between smoking and aortic aneurysm in men than in women, in older persons than in younger persons and in persons with normal weight than in those with obesity, but no difference was observed by prevalent hypertension. These subgroup analyses findings are partly in line with aortic aneurysm screening guidelines that emphasize older male smokers^32^, although it should be mentioned risk is considerably elevated across all subgroups of smokers as also discussed by others^33^.

Several biological mechanisms could explain the observed association between smoking and increased risk of aortic aneurysm. Smoking activates tissue plasminogen activators (tPA), which induce macrophages to produce matrix metalloproteinases (MMPs) and disrupt collagen synthesis, resulting in changes in normal medial artery structure, thus likely increasing the risk of aortic aneurysm^34–36^. At the same time, tPA may decompose normal lamellar elastin matrix, which is mediated by MMPs released by macrophages^34–37^. However, existing studies are inconsistent about the expression of MMPs. In a rat study, nicotine weakened vascular walls, increased gel breakdown activity, promoted the destruction of elastin and collagen in the abdominal aorta, and led to the up-regulation of MMP-12 expression^38^, while another study found no association between smoking-induced aortic dilation and MMP-9 and − 12 expression^39^. Other studies have found that both tobacco smoke and benzopyrene, a compound found in cigarette smoke, increase angiotensin-2-induced aortic muscle cell apoptosis, aortic macrophage infiltration, MMP-2, 9, and 12, and nuclear factor - B expression, and increase the risk of aortic aneurysm^40,41^.

Strengths of the study include (1) the prospective design, which avoids recall bias and reduces the possibility of selection bias, (2) the large sample size, which allowed us to investigate specific subsites within the aorta, (3) detailed assessment of other risk factors, which allowed us to adjust for a range of suspected or established risk factors, (4) linkage to health registries, and (5) the long follow-up, which resulted in an adequate number of cases and deaths. Self-reported information on smoking has been shown to be highly valid in validation studies, although some measurement error is possible. Given the strong associations observed between smoking and a large number of diseases and mortality outcomes in many studies^20,42^, we consider misclassification of smoking status less of a concern, although changes in smoking status over time could have led to conservative estimates of risk. Although the UK Biobank participants are not representative for the UK population because of low participation rates (5.5%) and the vast majority being of white ethnicity, the observed exposure-outcome relations are still likely to be valid. Further studies in more diverse populations could be informative. However, our findings are consistent with the results of a previous meta-analysis^6^, which showed similar associations across different geographic regions, suggesting our findings may be broadly generalizable. The number of participants lost to follow-up was low and is not likely to have substantially biased the results.

This study showed a strong positive association between smoking and the incidence and mortality from aortic aneurysm and provides further evidence consistent with a previous meta-analysis^6^. The study also provides further results demonstrating substantial reductions in risk with longer durations of smoking cessation and therefore provide important public health guidance for smoking cessation among current smokers, in addition to smoking prevention among non-smokers. Any further studies on aortic aneurysm subsites and subtypes could be informative.

In summary, we found strong positive associations between smoking and risk of aortic aneurysm with dose-response associations, which were most pronounced for abdominal, thoracoabdominal, and unspecified site aortic aneurysms, and largely null for thoracic aortic aneurysms. Longer durations of smoking cessation substantially reduced aortic aneurysm risk compared to current smokers, and with ≥ 30 years of smoking cessation risk appeared comparable to that of never smokers. These findings provide strong support for public health recommendations and policies for smoking prevention and cessation.

## Supplementary Information

Below is the link to the electronic supplementary material.

Supplementary Material 1

## Data Availability

The data for the current study is available for researchers at [https://ukbiobank.ac.uk].
