# Supplementary material for: Tobacco smoking and the risk of aortic aneurysm in the UK biobank
Source: Sci Rep. 2025 Sep 1;15:32191. doi: 10.1038/s41598-025-18013-x (PMC12402329; doi:10.1038/s41598-025-18013-x)
Supplement: Supplementary file 1 — Supplementary Material 1 [file 41598_2025_18013_MOESM1_ESM.pdf]

Supplement for:

Zheng L, Baroom G, Naqvi REZ, Heath AK, Berlanga-Taylor A, Hibino M, Aune D. Tobacco smoking and the risk of aortic aneurysm in the UK Biobank. Scientific Reports 2025 https://doi.org/10.1038/s41598-025-18013-x

Supplementary Table 1. Smoking status and aortic aneurysm in strata of age, sex, BMI and hypertension status

Smoking status			Never	Former	Current	$P_{interaction}$
Age	<60 years	HR (95% CI)	1.00	1.38 (1.17-1.63)	2.84 (2.37-3.41)	<0.001
	≥60 years	HR (95% CI)	1.00	1.85 (1.68-2.04)	5.13 (4.58-5.75)	
Sex	Men	HR (95% CI)	1.00	1.44 (1.21-1.71)	5.43 (4.47-6.60)	<0.001
	Women	HR (95% CI)	1.00	1.75 (1.59-1.92)	4.09 (3.67-4.57)	
BMI	<25	HR (95% CI)	1.00	1.50 (1.25-1.79)	5.39 (4.48-6.48)	0.002
	25-<30	HR (95% CI)	1.00	1.66 (1.47-1.86)	4.13 (3.58-4.75)	
	≥30	HR (95% CI)	1.00	1.84 (1.58-2.13)	3.81 (3.17-4.59)	
Hypertension	No	HR (95% CI)	1.00	1.73 (1.47-2.04)	4.45 (3.71-5.34)	0.89
	Yes	HR (95% CI)	1.00	1.68 (1.53-1.85)	4.32 (3.86-4.84)	

Adjusted for age, sex, ethnicity, Townsend deprivation index, education, BMI, height, leisure-time physical activity, history of connective tissue disease
